# The Lived Experiences of Individuals with Type 2 Diabetes Mellitus with Poor Glycaemic Control in Nigeria: A Qualitative Study

**DOI:** 10.1177/11795514251384044

**Published:** 2025-10-18

**Authors:** Oyedeji Ayodeji, Madeleine Benton, Lois Orton, Scott Weich

**Affiliations:** 1 School of Health and Related Research, The University of Sheffield, UK; 2 Department of Psychological Medicine, Institute of Psychiatry, Psychology, and Neuroscience, King’s College London, London, UK; 3 School of Sociological Studies, The University of Sheffield, UK

**Keywords:** type 2 diabetes, poor glycaemic control, lived experience, qualitative study, LMICs, Nigeria

## Abstract

**Background:**

Many individuals living with type 2 diabetes mellitus (T2DM) struggle to maintain optimal glycaemic control. Reports from Nigeria show particularly high rates of poor glycaemic control, increasing the risk of microvascular and macrovascular complications. Little research has explored the lived experiences of individuals living with T2DM with poor glycaemic control in Nigeria, particularly in secondary healthcare settings, to guide improvements in care.

**Objective:**

This study explored the experiences of individuals living with T2DM with poor glycaemic control.

**Method:**

A qualitative research design was used. Semi-structured, individual interviews were conducted with 14 participants, aged 35 to 74 years, recruited from 3 secondary healthcare institutions in Lagos, Nigeria.

**Results:**

Four key themes were generated: (1) Beyond the T2DM diagnosis, which captures the perceptions of T2DM, the financial burden of the condition, and the onset of physical health issues associated with T2DM; (2) Psychological impact of T2DM, which highlights mental health difficulties and experiences of stigma; (3) Managing and living with T2DM, which describes the use of traditional medicine, the influence of religious beliefs and the importance of community and social networks and (4) Diabetes care at secondary healthcare institutions, which highlights patient-provider interactions and the gaps in information and education.

**Conclusion:**

The findings provide valuable insight into the lived experiences of individuals with T2DM with poor glycaemic control and underscore the importance of addressing knowledge gaps and providing psychological support as integral components of comprehensive diabetes care.

## Plain Language Summary

Living with Type 2 Diabetes in Nigeria: Personal Experiences of People Who Face Challenges in Managing Their Blood Sugar

Why was the study done? Many people with type 2 diabetes (T2D) face challenges in managing their blood sugar levels. In Nigeria, this is a common issue that can result in significant health issues such as damage to the eyes, kidneys, nerves or heart. However, there is still limited information about the experiences of people in Nigeria who face challenges in managing their blood sugar levels, especially those that receive treatment at secondary care centres. Understanding their experiences can help improve the support and care they receive. What did the researchers do? This study explored what life is like for people living with T2D who face challenges in managing their blood sugar levels. Fourteen adults, aged 35 to 74, from three secondary health centres in Lagos, Nigeria shared their personal experiences. What did the researchers find? People shared their views about T2D, the costs involved in its treatment and the physical health problems that came with it. They spoke about how living with T2D affected their emotions, including feeling worried and judged by others. They shared how they coped with challenges in achieving good blood sugar control, including using traditional medicine, leaning on their faith and getting support from family and their community. Finally, they talked about their experiences with care at the secondary care centres, including their interactions with healthcare providers and the lack of clear information. What do the findings mean? This study helps us better understand the life of people with T2D who face challenges in managing their blood sugar levels. It shows the importance of providing people with more information about diabetes and emotional support as key parts of good diabetes care.

## Introduction

Type 2 diabetes mellitus (T2DM) is a prevalent, progressive, chronic condition associated with microvascular and macrovascular complications, which accounts for over 90% of diabetes cases globally.^1^ The International Diabetes Federation (IDF)^2^ estimates that 540 million people worldwide have diabetes, with 75% of these individuals living in low- and middle-income countries (LMICs) such as Nigeria. The IDF in its 2021 report, predicts an increase to 783 million by 2045, with 94% of this growth occurring in LMICs due to greater population increases.^2^

Managing T2DM can be challenging due to its dependence on individual lifestyle and daily self-management tasks, such as monitoring dietary intake, physical activity, carbohydrate consumption and regular testing of blood glucose levels. These tasks are crucial for maintaining optimal glycaemic control, defined as glycated haemoglobin (HbA1c) under 7%.^3^ While individual health behaviours play an important role in maintaining optimal glycaemic control, many people face significant challenges in meeting the demands of self-management.^4,5^ Additionally, a lack of knowledge and skills can lead to disengagement with self-care, ultimately resulting in poorer health outcomes.

Although poor glycaemic control is associated with an increased risk of microvascular and macrovascular complications, it remains a common challenge across various settings.^6-8^ Existing literature reports several factors as contributing to poor glycaemic control in individuals with T2DM. Within the Nigerian setting, studies have shown that T2DM is strongly associated with illness perception, knowledge and mental health problems like diabetes distress and depression.^9-11^ There is the need to better understand the experiences of individuals with poor glycaemic control in Nigeria, given the country’s high diabetes burden in the African region and the significant proportion of undiagnosed T2DM cases.^12^ Despite reports indicating high rates of poor glycaemic control (ranging from 40% to 74%) in this setting, existing studies^8,13,14^ exploring the experiences of individuals living with T2DM with poor glycaemic control have predominantly utilized quantitative approaches and been conducted within tertiary healthcare institutions. As a result, there is a paucity of evidence exploring patient experiences in secondary healthcare settings in Nigeria, where the majority receive care due to their widespread distribution and relative accessibility of these facilities across towns. Findings involving patient populations in secondary healthcare institutions will provide additional insight in understanding the experiences of individuals with T2DM with poor glycaemic control in this setting to guide improvements in care. Therefore, this research investigates the experiences of individuals living with T2DM with poor glycaemic control in secondary healthcare institutions in Lagos State, Nigeria.

## Methods

### Study Design and Setting

A qualitative study design using semi-structured interviews was conducted to explore the experiences of individuals living with T2DM with poor glycaemic control in Lagos, Nigeria. Lagos is the most populous urban state in Nigeria with a population of 17.5 million people and is home to diverse language groups.^15^ According to the Lagos State Ministry of Health,^16^ it was reported that Lagos has 30 registered secondary healthcare facilities that are grouped based on its 5 administrative regions: Ikeja, Badagry, Ikorodu, Lagos Island and Epe. These regions represent both urban (Ikeja, Lagos Island) and peri-urban (Ikorodu, Badagry and Epe) settings. For this study, 3 secondary healthcare facilities; General Hospital Gbagada (Ikeja), General Hospital Lagos Island (Lagos Island) and General Hospital Ikorodu (Ikorodu), were selected using convenience sampling. To mitigate potential bias associated with this sampling method, hospitals were selected from both urban and peri-urban regions to ensure diversity among patients in terms of socioeconomic characteristics and experiential contexts. In Nigeria, secondary healthcare facilities serve as the first point of contact for patients resulting in most individuals with T2DM receiving treatment at these facilities. Patients are routinely managed by the medical officers and specialist physicians (endocrinologists) with advanced knowledge and experience in managing T2DM. Their care is further supported by nurses and dietitians. The outpatient clinic at the selected hospitals runs twice a week, and the target population for this study was adult patients with T2DM attending these clinics.

### Recruitment

Potential participants were identified by healthcare professionals in 3 secondary healthcare facilities in Lagos. The study inclusion criteria were: aged 18 years and over, a confirmed diagnosis of T2DM and HbA1c higher than 7% (53 mmol/mol) for 6 months or longer. The definition of poor glycaemic control was in line with the guidelines of the American Diabetes Association.^3^ Pregnant women and patients with diabetes complications were excluded.

Potential participants meeting the inclusion criteria were invited by healthcare professionals to discuss the study with the researcher and were provided with a participant information sheet. Those interested and consenting participants provided their contact details and were contacted later by the researcher to confirm their participation, answer any questions and arrange a convenient date, time and venue for the interviews. Written consent was obtained by the first author (AO) before the interview took place. Interviews took place at venues that were convenient for the participants such as the diabetes clinics and participants’ homes with the single exception of a participant who requested to be interviewed over the telephone. Recruitment of new participants ended when data saturation was reached, that is, when sufficient understanding had been achieved and no key themes were emerging from the interviews.

### Procedure

The interview topic guide was developed based on the aims of the study in combination with previous literature on T2DM.^17,18^ Although pilot interviews were not conducted due to the COVID-19 pandemic, the interview guide was reviewed by the fourth author (SW) prior to the commencement of the study. Typical questions were: (1) “Can you tell me about the time you were diagnosed with T2DM?” (2) “Was there a time when things were going well in relation to managing your illness?” Further information is available in the supplemental materials. Probing questions (eg, How did you feel when you were diagnosed with diabetes? Why do you think they were going well?) were used to gain deeper understanding of participants experience. The interview guide was modified as the study progressed with the participants in terms of wording and phrasing of questions to eliminate questions that were identified as ambiguous. Participant recruitment and interviews were conducted between late September 2020 and April 2021. In addition to conducting the interviews in English, which is the official language in Nigeria, they were also conducted in Nigerian Pidgin English, which is a widely spoken language and acts as a bridge between different language groups.

### Data Analysis

All interviews were audio-recorded and transcribed verbatim by the first author (AO). Names of participants were replaced with identification codes to preserve anonymity. Interview transcripts were imported into qualitative software NVivo and analysed by the first author (AO) using thematic analysis.^19^ The process of analysis began with the reading and re-reading of the interview transcripts to become familiar with the data and check transcript accuracy against the audio recordings. Next, codes were generated utilizing the words said by participants in their interviews and these codes were then categorized into themes and sub-themes with relevant coded data extracts. These were then validated by SW, JC and MB. The themes were reviewed, and necessary amendments were made to the themes and sub-themes to ensure that they reflected the qualitative data.

## Results

### Participants

Twenty-four individuals who met the inclusion criteria were referred by healthcare professionals and thus were invited to participate in the study. Of these, 14 participants accepted to be interviewed for this study. Of the remaining individuals who met the inclusion criteria, n = 10 were no longer reachable via their contact number, n = 1 pre-consented to the interview but withdrew consent prior to commencement of the interview and n = 3 were interested but no suitable date could be found because of work commitments. The average duration of the interviews was 50 minutes. Out of the n = 14 participants aged between 35 and 74 years (mean age = 58 years), n = 6 were men and n = 8 were women. The duration of participants’ T2DM diagnosis ranged from 1 year to 31 years. Of the n = 14 participants, n = 8 participants were still employed, n = 3 were unemployed and n = 3 were retired. Furthermore, education levels of the participants were such that n = 6 participants attained tertiary education, n = 4 went to primary school, n = 2 attained secondary school education and n = 2 received no schooling/education. Majority of participants were married (n = 9), n = 1 was single and n = 4 were widowed. Seven of the participants had family history of T2DM and the remaining n = 7 had no family history of T2DM.

### Themes

Four themes each with between 2 and 3 sub-themes were generated. The 4 themes that were generated were: (i) *Beyond the T2DM diagnosis*, (ii) *Psychological impact of T2DM*, (iii) *Managing and living with T2DM*, and (iv) *Diabetes care at secondary healthcare institutions.* The themes and sub-themes are summarized in Table 1 and discussed across the remainder of this section.

**Table 1.** Summary of Themes and Sub-themes.

Themes	Sub-themes
Beyond the T2DM diagnosis	Personal perceptions and interpretations of T2DM
	Financial burden of the condition
	Initial and ongoing physical health issues
Psychological impact of T2DM	Mental health challenges
	Experienced stigma and its effect
Managing and living with T2DM	Use of traditional medicine practices for management.
	The role of religious beliefs.
	The importance of community and relationships
Diabetes care at secondary healthcare institutions	Patient-provider dynamics
	Information and education gaps

#### Theme 1: Beyond the T2DM Diagnosis

##### Sub Theme 1.1: Personal Perceptions and Interpretations of T2DM

Perception about diabetes and self-management plays a salient role in influencing patients’ health behaviours, which are essential for achieving and maintaining optimal glycaemic control. Most participants believed that T2DM and poor glycaemic control had explanations beyond those provided by medical professionals.

I knew it was sugar related problems and that was how I felt anyway I knew I would be treated I knew I would get out of it but I didn’t know it would continue in me but it continued until now (Participant 1, male 74 years)

I just believe that any sickness is from God Almighty. . .. (Participant 3, male, 35 years)

I always think that if it’s something they give me medicine for that is doing me, it would have come down but this one. . .I always take the medicine it will be going high, shey [is it not] that the person in the village is doing me this thing? Nothing that I don’t think of. . .I feel within myself that it is somebody that is doing me, that they sent this diabetes to me, for me to get it and die (Participant 13, female, 56 years)

One participant described diabetes as a foreigner that would soon leave her body, expressing hope and relief from the burdensome lifestyle constraints imposed by the diabetes self-management regimen. Another participant compared T2DM to a problem and felt that there must be a solution if 1 continues searching.

You know somebody will have health challenge, you will find a way yourself how it will go out. . .what kind of drugs can somebody take for this thing to go because it is not good. . .when you like eba (cassava flakes), when they [are] telling you not to eat the eba again, will you be happy? I will be happy if I don’t have it. . .because that particular disease I don’t want it to stay long. It [diabetes] is a foreign disease it’ll go out it will crash by fire by force (Participant 9, female, 60 years)

I believe that in the world problems are to be solved so if there is a problem you just have to look for solution and if you look for solution you are able to solve the problem and if you have not gotten it, keep on searching. . .until you get the solution you want (Participant 14, male, 62 years)

##### Sub Theme 1.2: Financial Burden of the Condition

The cost of diabetes care is high, with patients paying out of pocket for treatment services including medications. For some participants, the thought of how much is regularly spent on treating T2DM increased their stress levels and heightened their desire to be free of the illness, whereas other participants reported that the poor efficacy of locally available medications forced them to buy more expensive alternatives from overseas.

Yes, I wish I didn’t have it because the money I spend in getting the drugs at least every month I buy drugs worth #15,000 (GBP20.83) (Participant 2, female, 72 years)

. . .I am not happy with it. . .because it causes me to spend so much money (Participant 11, female, 64 years)

##### Sub Theme 1.3: Initial and Ongoing Physical Health Issues

Some participants stated that they had fewer medical complaints before diabetes diagnosis but that has changed as T2DM has had a considerable impact on their sight, sexuality and upper and lower limbs.

It has stopped my sex life my [manly part] can never get up again yes, I am coming to know that I cannot make love again to a woman. . . (Participant 6, male, 64 years)

It is just diabetes alone I don’t have any other illness. When it [diabetes] came is when my leg started hurting. . .before it came I don’t get tired (Participant 8, female, 64 years)

Both of my leg pains me, it does me to the level that I don’t know what is going on, to the level that diabetes makes it impossible for me to use my hand to collect something. . .it doesn’t feel like my hand, I cannot use it to carry a bucket to bathe unless my children carry it for me (Participant 13, female, 56 years)

#### Theme 2: Psychological Impact of T2DM

##### Sub Theme 2.1: Mental Health Challenges

The results point to the fact that most participants with poorly controlled T2DM experienced mental health problems. Self-management was described as overwhelming and fatiguing by participants and they reported experiencing a range of thoughts and feelings including anger, frustration, sadness and helplessness as they struggled to understand why their self-management efforts did not culminate in better control of the illness.

Living with diabetes is like you have a demon in you, you have to constantly suppress and fight, so diabetes is like that, it’s frustrating, very frustrating (Participant 12, female, 52 years)

Some participants reported feeling sad, frustrated and unhappy about lifestyle restrictions, such as having to give up the food they loved, which affected their motivation to adhere to self-management guidelines.

You know this thing is a routine. . .that you’ll do it [self-management] every day, you will be bored at times I’ll just be tired of taking drugs (Participant 7, male, 45 years)

Your favorite food they ask you not to take again. . .you will not be happy, you will be feeling sad all the time. . . (Participant 9, female, 60 years)

One participant expressed feelings of severe emotional distress and hopeless thought patterns arising from the lack of improvement in her blood sugar levels, despite consistently taking her medications. This sense of hopelessness culminated in suicidal ideation and a short-lived decision to stop using the medication out of frustration and despair. Her experiences highlight how difficulties with glycaemic control can impair emotional resilience and the will to live.

What is causing the sugar level to go high as I always take medicine every day by day, there is no day I don’t take! That’s why if I think. . .I’ll just be down, I won’t be able to talk, even if anybody talks to me I won’t be able to answer them, I will just be quiet there, that’s how I feel, I will just be down, even my daughter will talk to me, I’ll just say, please leave me, I don’t want to talk (Participant 13, female, 56 years)

. . .I don’t know myself again, it makes me to feel bad, I don’t feel happy again that I’m living. The sickness didn’t let me get up again, it didn’t let me sit down! I angrily said I wouldn’t take any medicine again, if it wants to kill me, let it kill me. I don’t want, I always take the medicine, [and] instead of the thing to get better, it worsens (Participant 13, female, 56 years)

##### Sub Theme 2.2: Experienced Stigma and Its Effect

Participants recounted feelings of internalized stigma specific to their condition. They felt that they could not tell others about their burden because of mistrust and the fear of being rejected, which was noted as having a negative impact on their emotional wellbeing and contributing to poor mental health.

You’re not able to communicate, you don’t understand what’s happening in your body, you are afraid to tell people that this thing is killing you so that they don’t reject you but indirectly you are withdrawn. . .and you are getting depressed, and the damage is increasing (Participant 12, female, 52 years)

One participant stated that her husband was the only person she could confide in about her experiences, as telling others, such as friends, would subject her to ridicule.

No [. . .], I’m not sharing my feelings with anybody it’s only my husband. You don’t know your friend, you don’t know who and who is your friend. *One* day you will not be around they will sit down together and gather themselves together and they laugh. . . (Participant 9, female, 60 years)

Stigma from the community, including friends, was also disconcerting for participants, who expressed frustration at the verbal remarks and language used when shopping for daily food or honouring invitation to social gatherings.

No food is diabetic food, no drink is diabetic drink it’s annoying. . .people don’t want to be pictured that way, people don’t want to be seen that way we just want to feel normal why are you telling me this the diabetic. I know all my life I am living with a condition that I’m going to live with the rest of my life so why are you labelling me that (Participant 12, female, 52 years)

#### Theme 3: Managing and Living with T2DM

##### Sub Theme 3.1: Use of Traditional Medicine Practices for Management

The high cost of prescribed medications led participants to seek cheaper treatment methods, such as traditional medicines. These were sometimes used in combination with prescribed medication, and many participants reported that the traditional medicines helped reduce their blood sugar levels and improved their sexual function.

I still take it. . . it’s scent leaf I know it’s very effective if I take scent leaf now it will crash my sugar level. . . (Participant 6, male, 64 years)

It is only when I wanted to have sex, I saw that I couldn’t do unlike before, so there was a time I used a herbal something and the thing picked up. . .yes, it helped (Participant 14, male, 62 years)

##### Sub Theme 3.2: The Role of Religious Beliefs

Some participants with either Christian or Islamic faith highlighted that although living with diabetes is unpleasant, their religious faith and prayers were helping them effectively manage their T2DM as well as the emotional burden associated with living with the condition.

. . .faith in God has really affected me positively and has really helped me not to panic, not to show much fear if any at all, so it is that faith that really helped me. . .it is a bad thing to live with diabetes, however if you have faith in God and you have experienced God in your life, it will help you to have a better opinion, if not, *one* will be living in fear (Participant 14, male, 62 years)

Prayer was considered as a better option in relation to talking about the burden of the disease than talking about it to others.

. . .I don’t talk to anybody about myself, but I talk with my God, it’s only God that can do anything because our God is able to take care of me. . . (Participant 9, female, 60 years)

##### Sub Theme 3.3: The Importance of Community and Relationships

Participants reported that family members, including children, play crucial roles in supporting managing their condition by encouraging adherence to their diet regimen and supporting their psychological wellbeing.

Sometimes if I want to eat something that I’m craving for that they know will boost the sugar, they will say, daddy this *one* is not good for you to eat, I will say okay I will stop (Participant 5, male, 60 years)

While some of the participants received assistance from family members, a few others reported receiving recommendations from friends to experiment with natural remedies to help treat their illness.

My friend advised [me] on herbal drug like bitter leaf and scent leaf that if I took bitter leaf and scent leaf it’ll go so, I started drinking bitter leaf and scent leaf but it never went (Participant 6, male, 64 years)

Participants conveyed the need for greater peer support from individuals living with T2DM as it presented an opportunity to receive help from individuals who have experienced similar struggles. It was also identified as being beneficial for the emotional wellbeing of persons living with diabetes.

When you now see people that have a similar [condition] and are in a similar situation like you. . .and they are living fine if they don’t even tell you, you won’t know that they have it. . .it gives you hope (Participant 7, male, 45 years)

#### Theme 4: Diabetes Care at Secondary Healthcare Institutions

##### Sub Theme 4.1: Patient-Provider Dynamics

Participants reported that healthcare professionals’ responses to their struggles in achieving optimal glycaemic control were centred around prescribing medication without exploring other contributory factors, embodying a passive patient-doctor relationship within a medical model of care. This model of care is exemplified by some participants’ narratives that they did not receive adequate emotional support from healthcare professionals in their efforts to control their blood sugar.

Once I explain how I am they’ll write drugs for me whether the ones they want to add to my current drugs or the ones they want to remove (Participant 2, female, 72 years)

They know that the blood sugar is high, but they don’t ask me how I feel how I think about the diabetes or something (Participant 3, male, 35 years)

. . .when you talk to all these doctors if you are living in Lagos you’ll know the traffic situation and what somebody that is supposed to see it from your own perspective is still telling you that because you didn’t do this. You know it pains if this person is inside my shoes this person should just put herself in my shoe and know how it feels (Participant 7, male, 45 years)

One participant stated that some of the healthcare professionals did not have the patients’ best interests and were only interested in financial benefits.

The doctors don’t have our interest. Most of them don’t have our interest they only think of their pocket. . . (Participant 12, female, 52 years)

##### Sub Theme 4.2: Information and Education Gaps

Participants reported inadequate information and education about diabetes within the healthcare system, which intensified their anxieties about living with the condition. Some of the available information was from different settings and as such, were not considered relevant to them.

For me I feel that there’s not enough information anywhere. Whatever website you go [to] now is what is applicable abroad [and] we don’t really have enough information on the kind of foods we can combine here that can help us live a healthier life (Participant 2, female, 72 years)

## Discussion

This study aimed to explore the experiences of individuals living with T2DM with poor glycaemic control in Lagos State, Nigeria. The findings suggest that optimal glycaemic control is influenced by multiple factors, including, diabetes-related knowledge, financial support, psychological well-being, social support and the patient-provider relationship.

Participants in this study used metaphors to describe the challenges of controlling diabetes, often expressing a sense of helplessness. This language contrasts with the conventional biomedical understanding that type 2 diabetes can be managed through daily medication and lifestyle changes such as physical activity. The use of metaphors to understand illness perception has been explored in previous studies. For instance, it was found that Congolese patients used metaphors to express beliefs about the extent of personal control over diabetes, revealing culturally embedded understandings of responsibility and attributions.^20^

Literature has emphasized the impact of illness beliefs on self-management behaviour, particularly when individuals perceive themselves as unable to change negative outcomes.^21,22^ According to Biber et al,^23^ as cited in Connor et al,^24^ metaphors that conceptualize diabetes as a stranger/foreigner or brought on by luck/chance and God, will reflect an external orientation. This orientation suggests that these individuals do not view themselves as having a role in the onset or control of the illness. Haskas et al^25^ further argue that individuals with such external beliefs are more likely to engage in unhealthy practices, which may explain the use of traditional medicine by participants in the present study. In contrast, individuals with an internal orientation tend to view diabetes as a condition they can influence and manage, often using metaphors that reflect responsibility and agency.

Financial constraints were notable, as most persons living with diabetes must pay out of pocket for medical care, in addition to facing the high cost of healthy foods. This challenge was pervasive among participants in this study, even those who were economically advantaged, and was observed to influence the use of traditional medicine in this population. While living with diabetes was identified as a significant stressor contributing to poor mental health, previous studies^26,27^ have also reported a cyclical and mutually reinforcing relationship between financial hardship and poor mental health in resource-limited countries. This effect may be further pronounced in Nigeria, where 40% of the population lives in povert.^27,28^

Living with diabetes is also physically challenging with participants reporting the impact on their day-to-day life. Although this was not a common complaint among participants, the study documented the challenges to physical health experienced by participants, with the illness affecting their sight, sexual drive and limbs. This is comparable with research in Zambia that also found physical health issues such as sexual dysfunction, poor sight and numbness in limbs to be among the experiences of living with diabetes.^29^ However, it should be noted that in this present study, sexual dysfunction was commonly reported by male participants, and it is important to explore if such concern is also shared by female participants as it has been previously noted that female sexual dysfunction is more difficult to define.^30^

Majority of the participants acknowledged experiencing mental health challenges resulting from the burden of living with T2DM. Feelings of frustration, tiredness and helplessness were commonly reported and attributed to the illness. Poor psychological health also appeared to hinder consistent self-monitoring of blood glucose levels. These findings are consistent with research in Nigeria and Zambia, which similarly documented significant mental health struggles among individuals living with T2DM.^14,29^ In the current study, the lack of integrated mental health support in diabetes care may have worsened these emotional challenges, with some participants expressing suicidal thoughts and a preference for death over continued living.

These findings revealed that experiences of stigma from friends and healthcare professionals contributed to poor mental health, non-disclosure of the disease and reluctance to seek help. This highlights type 2 diabetes mellitus (T2DM) as a stigma-related illness, as previously suggested by Potter et al^31^ and Winkley et al.^32^ Such stigma may stem from a lack of public health education, misconceptions and negative beliefs about diabetes, particularly given that health information is primarily disseminated through clinics and healthcare professionals.^33,34^

Individuals living with T2DM with poor glycaemic control utilized different coping strategies to buffer the stresses associated with their illness. Religious faith and social support were reported as being instrumental in motivating them to take charge of their health and improve engagement with self-management practices to minimize the impact of the illness.^35^ Nigeria is renowned for being a nation with strong religious and societal values which are important for outlook on life and influential on health behaviours and choices. Even though religious faith can induce frustrations when expected healing does not occur, it has been reported to have psychosocial benefits and improve physical functioning for patients.^36^ The illness experience of T2DM is often described as stressful. In this study, participants’ use of religious faith as a coping mechanism reflects findings from other qualitative studies conducted among Ghanaian and Indonesian patients.^37,38^

The use of traditional medicine among individuals living with T2DM is a common practice in Nigeria, as noted in previous studies,^39,40^ where it is used to manage chronic conditions and promote general well-being. In the current study, participants cited its affordability as a key reason for its uptake. This finding aligns with research from Tanzania and Malaysia, where individuals with T2DM reported using traditional medicine to support diabetes care due to its accessibility and relatively low cost.^17,41^ Although Nigeria is among the few countries to formally recognize traditional medicine in alignment with the WHO’s Traditional Medicine Strategy, further research is needed to investigate potential interactions with prescribed medications, as the therapeutic mechanisms of many traditional remedies remain undefined.^42^

The patient-doctor relationship has been noted as being significant in diabetes care and findings from this study suggests that persons living with diabetes had a passive relationship with their healthcare professionals. This is consistent with the finding from previous systematic review in the Nigerian setting that reported the lack of partnership as patients are expected to act in accordance with healthcare professionals’ recommendations incontestably.^43^ This is likely due to patients’ limited access to relevant knowledge about diabetes and standard diabetes care which leads them rely on healthcare professionals to manage their condition. Furthermore, the lack of empathy from healthcare professionals regarding adjustment to new lifestyle as described by participants, has also been reported as a consistent challenge in persons with T2DM by Matthews et al.^44^ In our study, participants felt this also contributed to their distress and stigma, further illustrating the need for improvement of the healthcare service as well as a healthy patient-healthcare professional relationship.

The mean age of the participants in this study was 58 years, highlighting a predominantly middle-aged sample, consistent with similar studies in this setting.^14^ However, there are important limitations to consider. Firstly, pilot testing of the interview guide was not conducted due to COVID-19 restrictions, which may have limited opportunities to refine questions prior to data collection. Given that participants were recruited from 3 secondary healthcare facilities, the findings cannot be generalized to other secondary healthcare facilities within the state. Additionally, the use of a convenience sample excluded participants from other parts of Lagos, potentially omitting perspectives different from those included in the study. Lastly, allowing participants to speak in their native languages might have made them feel more comfortable and encouraged more open discussion, thereby enriching the data. However, this approach could have introduced selection bias, as the researcher may have been inclined to recruit only participants who shared their native language. To avoid this bias and address potential language proficiency challenges among participants not fluent in English, Nigerian Pidgin English was chosen as a suitable alternative, given its wide usage and comprehension across various language groups in the country.

## Conclusion

In conclusion, these findings provide valuable insights into the experiences of individuals with T2DM with poor glycaemic control in Nigeria, especially regarding mental health difficulties that are often overlooked in diabetes care. By contributing to the growing body of literature on T2DM in LMICs, the study highlights the need for an approach that incorporates psychological support into routine diabetes care. Evidence-based approaches such as diabetes education, cognitive behavioural therapy, which helps patients address maladaptive perceptions about T2DM and self-care behaviours and motivational interviewing, which supports behaviour change through goal-oriented discussions, could be adapted to address patients’ cognitive and emotional challenges. However, implementation may be limited by resource constraints, such as the shortage of trained mental health professionals, which is common in Nigeria and similar LMICs. Future service development and evaluative research should explore reallocation of provision of psychological support to trained non-mental health professionals such as peers. This approach may help overcome barriers and improve outcomes for individuals with poorly controlled T2DM.

## Supplemental Material

sj-doc-2-end-10.1177_11795514251384044 – Supplemental material for The Lived Experiences of Individuals with Type 2 Diabetes Mellitus with Poor Glycaemic Control in Nigeria: A Qualitative Study Supplemental material, sj-doc-2-end-10.1177_11795514251384044 for The Lived Experiences of Individuals with Type 2 Diabetes Mellitus with Poor Glycaemic Control in Nigeria: A Qualitative Study by Oyedeji Ayodeji, Madeleine Benton, Lois Orton and Scott Weich in Clinical Medicine Insights: Endocrinology and Diabetes

sj-docx-1-end-10.1177_11795514251384044 – Supplemental material for The Lived Experiences of Individuals with Type 2 Diabetes Mellitus with Poor Glycaemic Control in Nigeria: A Qualitative Study Supplemental material, sj-docx-1-end-10.1177_11795514251384044 for The Lived Experiences of Individuals with Type 2 Diabetes Mellitus with Poor Glycaemic Control in Nigeria: A Qualitative Study by Oyedeji Ayodeji, Madeleine Benton, Lois Orton and Scott Weich in Clinical Medicine Insights: Endocrinology and Diabetes

## References

[1] World Health Organization (WHO). Fact sheet diabetes. 2021. Accessed 19 July 2024. https://www.who.int/news-room/fact-sheets/detail/diabetes

[2] International Diabetes Federation. IDF diabetes atlas - 10th edition. 2021. Accessed 19 July 2024. https://diabetesatlas.org/key-messages.html 35914061

[3] American Diabetes Association. 6. Glycemic targets: standards of medical care in diabetes-2020. Diabetes Care. 2020;43(Suppl 1):S66-S76. 10.2337/dc20-S006 31862749

[4] Fisher L Gonzalez JS Polonsky WH. The confusing tale of depression and distress in patients with diabetes: a call for greater clarity and precision. Diabetic Med. 2014;31(7):764-772. 24606397 10.1111/dme.12428 PMC4065190

[5] Stephani V Opoku D Beran D. Self-management of diabetes in Sub-Saharan Africa: a systematic review. BMC Public Health. 2018;18(1):1148. 30268115 10.1186/s12889-018-6050-0 PMC6162903

[6] Alzaheb RA Altemani AH. The prevalence and determinants of poor glycemic control among adults with type 2 diabetes mellitus in Saudi Arabia. Diabetes Metab Syndr Obes. 2018;11:15-21. 29430192 10.2147/DMSO.S156214 PMC5797462

[bibr7-11795514251384044] Omar SM Musa IR ElSouli A Adam I. Prevalence, risk factors, and glycaemic control of type 2 diabetes mellitus in eastern Sudan: a community-based study. Ther Adv Endocrinol Metab. 2019;10:2042018819860071. 10.1177/2042018819860071 PMC6598316 31275546

[8] Yakubu A Dahiru S Mainasara AS , et al Determinants of poor glycaemic control among type 2 diabetic patients at a suburban tertiary hospital in north-Western Nigeria. Int J Sci Health Res. 2020;5(4):207-214.

[9] Anakwue RC Young EE Ezenduka CC , et al Assessment of patients knowledge and attitude towards diabetes and its relationship with glycemic control: a cross-sectional study in a Nigerian tertiary hospital. Niger J Med. 2019;28(1):46-55.

[bibr10-11795514251384044] Asibong UE Okokon IB Ottah UN , et al Assessment of illness experience of type-2 diabetic patients using patient-centered care at Family Medicine Clinic in a Tertiary Hospital in Southern Nigeria. Int J Res. 2021;6(8):35-43.

[11] Ogbera A Adeyemi-Doro A. Emotional distress is associated with poor self care in type 2 diabetes mellitus. J Diabetes. 2011;3(4):348-352. 21883978 10.1111/j.1753-0407.2011.00156.x

[12] Olamoyegun MA Alare K Afolabi SA Aderinto N Adeyemi T. A systematic review and meta-analysis of the prevalence and risk factors of type 2 diabetes mellitus in Nigeria. Clin Diabetes Endocrinol. 2024;10(1):43. 39639395 10.1186/s40842-024-00209-1 PMC11622640

[13] Ibrahim AO Agboola SM Elegbede OT Ismail WO Agbesanwa TA Omolayo TA. Glycemic control and its association with sociodemographics, comorbid conditions, and medication adherence among patients with type 2 diabetes in southwestern Nigeria. J Int Med Res. 2021;49(10):3000605211044040. 10.1177/03000605211044040 PMC8504241 34632841

[14] Okurumeh AI Akpor OA Okeya OE Akpor OB. Type 2 diabetes mellitus patients’ lived experience at a tertiary hospital in Ekiti State, Nigeria. Sci Rep. 2022;12(1):8481. 35590021 10.1038/s41598-022-12633-3 PMC9120021

[15] Lagos Bureau of Statistics. Ministry of Economic Planning and Budget Abstract of local government statistics. 2020. Accessed 23 August 2024. http://mepb.lagosstate.gov.ng/lbs-publication/

[16] Lagos State Ministry of Health. Bridging Primary Care and Specialized Services. 2022. Accessed 23 August 2024. https://lagosministryofhealth.org/secondary-health-facilities/

[17] Mohamed AM Winkley-Bryant K Ismail K. The Development and Feasibility of a Culturally Tailored Malaysian Diabetes Education Intervention Using Motivational Interviewing (MY DEUMI) for People Newly Diagnosed with Type 2 Diabetes Mellitus. King’s College London; 2019.

[18] Perrin NED Khunti K Robertson N , et al Diabetes, depression and distress: the 3D-study: an explorative study to inform practice in the identification and management of depression and/or diabetes-specific distress in people with type 2 diabetes. University of Leicester; 2017.

[19] Clarke V Braun V. Teaching thematic analysis: overcoming challenges and developing strategies for effective learning. Psychologist. 2013;26(2):120-123.

[20] Lubaki FJP Francis JM Omole OB . Lived experiences and perspectives of patients with type 2 diabetes on poor glycaemic control in Kinshasa, Democratic Republic of Congo: a qualitative study, PREPRINT (Version 1). 2023. doi:10.21203/rs.3.rs-2365142/v1

[21] Vedhara K Dawe K Wetherell MA , et al Illness beliefs predict self-care behaviours in patients with diabetic foot ulcers: a prospective study. Diabetes Res Clin Pract. 2014;106(1):67-72. 25112923 10.1016/j.diabres.2014.07.018

[22] Widayanti AW Heydon S Norris P Green JA. Lay perceptions and illness experiences of people with type 2 diabetes in Indonesia: a qualitative study. Health Psychol Behav Med. 2019;8(1):1-15. 34040859 10.1080/21642850.2019.1699101 PMC8114388

[23] Biber D Johansson S Leech G , et al Longman Grammar of Spoken and Written English. Pearson Education; 1999.

[24] Connor U Antón M Goering E , et al Listening to patients’ voices: linguistic indicators related to diabetes self-management. Commun Med. 2013;9(1):1-12. 23763232

[25] Haskas Y Suarnianti S Angriani S , et al Impact of external locus of control on quality of life in patients with type 2 diabetes mellitus. Published online July 8, 2020. 10.21203/rs.3.rs-17733/v1

[26] Haushofer J Fehr E. On the psychology of poverty. Science. 2014;344(6186):862-867. 24855262 10.1126/science.1232491

[27] Lund C De Silva M Plagerson S , et al Poverty and mental disorders: breaking the cycle in low-income and middle-income countries. Lancet. 2011;378:1502-1514. 22008425 10.1016/S0140-6736(11)60754-X

[28] National Bureau of Statistics. Poverty in Nigeria 2019: measurement and estimates. 2020. Accessed 23 May 2022. https://www.nigerianstat.gov.ng/elibrary/read/1092

[29] Mwila KF Bwembya PA Jacobs C. Experiences and challenges of adults living with type 2 diabetes mellitus presenting at the university teaching hospital in Lusaka, Zambia. BMJ Open Diabetes Res Care. 2019;7:497. 10.1136/bmjdrc-2017-000497 PMC6861007 31798889

[30] Jackson G. Sexual dysfunction and diabetes. Int J Clin Pract. 2004;58:358-362. 15161120 10.1111/j.1368-5031.2004.00180.x

[31] Potter L Wallston K Trief P Ulbrecht J Juth V Smyth J. Attributing discrimination to weight: associations with well-being, self-care, and disease status in patients with type 2 diabetes mellitus. J Behav Med. 2015;38(6):863-875. 26133488 10.1007/s10865-015-9655-0 PMC4628883

[32] Winkley K Evwierhoma C Amiel SA Lempp HK Ismail K Forbes A. Patient explanations for non-attendance at structured diabetes education sessions for newly diagnosed type 2 diabetes: a qualitative study. Diabet Med. 2015;32(1):120-128. 25081181 10.1111/dme.12556

[33] Abdoli S Doosti Irani M Hardy LR Funnell M. A discussion paper on stigmatizing features of diabetes. Nurs Open. 2018;5(2):113-119. 29599986 10.1002/nop2.112 PMC5867293

[34] Brown K Avis M Hubbard M. Health beliefs of African-Caribbean people with Type 2 diabetes: a qualitative study. Br J Gen Pract. 2007;57(539):461-469. 17550671 PMC2078187

[35] Pesantes MA Del Valle A Diez-Canseco F , et al Family support and diabetes: patient’s experiences from a public hospital in Peru. Qual Health Res. 2018;28(12):1871-1882. 30066604 10.1177/1049732318784906 PMC6346298

[36] Simão TP Caldeira S De Carvalho EC. The effect of prayer on patients’ health: systematic literature review. Religion. 2016;7(1):11.

[37] de-Graft Aikins A Dodoo F Awuah RB , et al Knowledge and perceptions of type 2 diabetes among Ghanaian migrants in three European countries and Ghanaians in rural and urban Ghana: the RODAM qualitative study. PLoS One. 2019;14(4):e0214501. 10.1371/journal.pone.0214501 PMC6445464 30939148

[38] Arifin B Probandari A Purba AKR , et al Diabetes is a gift from god’ a qualitative study coping with diabetes distress by Indonesian outpatients. Qual Life Res. 2019;29(1):109-125. 31549366 10.1007/s11136-019-02299-2 PMC6962255

[39] Awodele O Osuolale JA. Medication adherence in type 2 diabetes patients: study of patients in Alimosho general hospital, Igando, Lagos, Nigeria. Afr Health Sci. 2015;15(2):513-522. 26124798 10.4314/ahs.v15i2.26 PMC4480454

[40] Ogundele SO Dada AO Mosuro OR. Clinical profile, knowledge, and beliefs about diabetes among patients attending a Tertiary Health Centre in Lagos: a cross-sectional survey. Niger J Clin Pract. 2016;19(4):508-512. 27251969 10.4103/1119-3077.183303

[41] Kolling M Winkley K von Deden M. “For someone who’s rich, it’s not a problem”. Insights from Tanzania on diabetes health-seeking and medical pluralism among dar es Salaam’s urban poor. Global Health. 2010;6(1):9. 20441575 10.1186/1744-8603-6-8 PMC2874526

[42] Chikezie PC Ojiako OA Nwufo KC. Overview of anti-diabetic medicinal plants: the Nigerian research experience. J Diabetes Metabol. 2015;06(06):546-552.

[43] Hashim ZP. The health beliefs and perceptions of adults living with diabetes type 2 in Nigeria. Eur J Med Health Sci. 2020;2(5):1-9. doi:10.24018/ejmed.2020.2.5.468

[44] Matthews SM Peden AR Rowles GD. Patient-provider communication: understanding diabetes management among adult females. Patient Educ Couns. 2009;76(1):31-37. 19157762 10.1016/j.pec.2008.11.022

