# Supplementary material for: The Lived Experiences of Individuals with Type 2 Diabetes Mellitus with Poor Glycaemic Control in Nigeria: A Qualitative Study
Source: Clin Med Insights Endocrinol Diabetes. 2025 Oct 18;18:11795514251384044. doi: 10.1177/11795514251384044 (PMC12547145; doi:10.1177/11795514251384044)
Supplement: sj-docx-1-end-10.1177_11795514251384044 – Supplemental material for The Lived Experiences of Individuals with Type 2 Diabetes Mellitus with Poor Glycaemic Control in Nigeria: A Qualitative Study [file sj-docx-1-end-10.1177_11795514251384044.docx]

**Supplementary Materials**

The interview guide consists of the following questions:

1. Diabetes beliefs

*Can you tell me about the time you were diagnosed with type 2 diabetes? How did you feel?*

*What did you know about diabetes before you were diagnosed?*

*How long have you been living with diabetes?*

*What do you think caused your diabetes?*

*Are you aware that diabetes is a life-long illness?*

*What are those things that your doctor tells you to do so that you can manage your blood sugar properly? How do you feel about them?*

*Is there anything that makes it difficult not to follow your doctor’s advice in managing your diabetes? What do you do about them?*

*How worried are you when your blood sugar level is high?*

*Apart from the doctor telling you to take your medicine to help control your blood sugar level, what other treatments do you use to control your blood sugar?*

1. Mental Health

*Do you wish that you didn’t have diabetes?*

*How would you describe your life right now as someone living with diabetes?*

*Are there activities that you used to do that you can’t do anymore that is as a result of the diabetes? How do you feel about them/this?*

*How is your relationship like with friends and family now that you have diabetes compared to before? How do you feel about them?*

*What do you think is different about your life compared to those who don’t have diabetes?*

*What aspect of living with diabetes has been challenging?*

*Are there other things you wish to add about living with diabetes?*

*Was there a time when things were going well in relation to managing your diabetes? Why do you think they were going well?*

*Was there something really useful maybe from family or friends or professionals that really made a difference?*
